# Condition dependence of male and female reproductive success: insights from a simultaneous hermaphrodite

**DOI:** 10.1002/ece3.1916

**Published:** 2016-01-18

**Authors:** Tim Janicke, Elodie Chapuis

**Affiliations:** ^1^ Centre d'Ecologie Fonctionnelle et EvolutiveUMR 5175CNRSUniversité de MontpellierUniversité Paul‐Valéry MontpellierEcole Pratique des Hautes EtudesMontpellier Cedex 05France; ^2^ Institut de recherche pour le développementUMR IPME (IRD, Université de Montpellier, CIRAD)911 avenue AgropolisBP 6450134394Montpellier Cedex 5France; ^3^ UMR “Peuplements Végétaux et Bio‐agresseurs en Milieu Tropical”CIRAD‐3P7 Chemin de l'IRATLigne Paradis97410Saint PierreLa RéunionFrance

**Keywords:** Condition, food availability, genic capture, mating behavior, sex allocation, sexual selection, simultaneous hermaphrodites

## Abstract

Sexually selected traits are predicted to show condition dependence by capturing the genetic quality of its bearer. In separate‐sexed organisms, this will ultimately translate into condition dependence of reproductive success of the sex that experiences sexual selection, which is typically the male. Such condition dependence of reproductive success is predicted to be higher in males than females under conditions promoting intense sexual selection. For simultaneous hermaphrodites, however, sex allocation theory predicts that individuals in poor condition channel relatively more resources into the male sex function at the expense of the female function. Thus, male reproductive success is expected to be less condition dependent than female reproductive success. We subjected individuals of the simultaneously hermaphroditic snail *Physa acuta* to two feeding treatments to test for condition dependence of male and female reproductive success under varying levels of male–male competition. Condition dependence was found for female, but not for male, reproductive success, meaning that selection on condition is relatively stronger through the female sex function. This effect was consistent over both male–male competition treatments. Decomposition of male and female reproductive performance revealed that individuals in poor condition copulated more in their male role, indicating an increased male allocation to mate acquisition. These findings suggest that sex‐specific condition dependence of reproductive success is at least partially driven by condition‐dependent sex allocation. We discuss the implications of condition‐dependent sex allocation for the evolution of sexually selected traits in simultaneous hermaphrodites.

## Introduction

The reproductive performance of an organism is typically governed by the acquisition of nutritional resources and the allocation of these resources to fitness‐related traits (Morehouse et al. 2010). For separate‐sexed organisms, there is a large body of empirical studies demonstrating that the reproductive success of males and females is positively affected by the individual's pool of resources that can be allocated to growth, survival, and reproduction, which has been termed the individual's condition (Rowe and Houle 1996; Morehouse 2014). In females, such condition dependence of reproductive success is thought to be direct consequence of energetically demanding production of nutrient‐rich eggs and/or the provision of postzygotic investment. By contrast, males of many species show condition‐dependent reproductive success, even though they typically invest very little resources per produced gamete and often provide less parental care than females (Lessells et al. 2009; Kokko and Jennions 2012). This is because sexual selection (defined as selection arising from competition for mates and their gametes sensu Shuker 2010) is predicted to select on condition‐dependent sexually selected traits such that the male's reproductive performance is tightly linked to its condition. Specifically, sexual selection theory predicts that sexually selected traits are costly to produce (Zahavi 1975) and therefore capture the individual's genetic quality at virtually all loci of genome (Rowe and Houle 1996) – also known as the “genic capture hypothesis” (Tomkins et al. 2004). This implies that sexual selection selects on loci underlying condition that are also subject to natural selection (defined as all nonsexual selection including selection on viability and fecundity sensu Endler 1986), which eventually leads to an alignment of sexual and natural selection to purge deleterious alleles across the genome (Whitlock and Agrawal 2009; Holman and Kokko 2013).

In support of this theory, there is ample empirical evidence showing that sexually selected traits exhibit condition dependence (reviewed in Cotton et al. 2004). This includes traits that are subject to precopulatory male–male competition (e.g., Emlen 1994) and female choice (e.g., David et al. 2000) as well as traits that are selected by postcopulatory episodes of selection such as testis size (Schulte‐Hostedde et al. 2005), sperm production rate (O'Dea et al. 2014), and genital morphology (Soto et al. 2007). Condition dependence of sexually selected traits will eventually result in condition dependence of male reproductive success (e.g., Fedina and Lewis 2006; Fricke et al. 2008; Fritzsche and Arnqvist 2015) and its magnitude is predicted to reflect the intensity of sexual selection (Johnstone et al. 2009). Selection on condition may even be stronger in males compared with females whenever sexual selection on condition‐dependent traits is strong enough to impose a higher net selection on males than does natural selection on females. However, very few attempts have been made to test for sex‐specific condition dependence of reproductive success. Zikovitz and Agrawal (2013) exposed males and females of the fruit fly *Drosophila melanogaster* to different feeding regimes and tested for sex‐specific selection on condition under varying levels of male–male competition. Their results suggest that reproductive success is more condition dependent in males than females, but only in the context of intense male–male competition (Zikovitz and Agrawal 2013).

For simultaneous hermaphrodites (hereafter called hermaphrodites), we also expect that reproductive success is condition dependent. However, hermaphrodites possess an additional route to fitness compared with separate‐sexed organisms (Michiels 1998; Schärer et al. 2015), which has important implications for sex‐specific condition dependence of reproductive success. More precisely, hermaphrodites have the unique opportunity to allocate resources toward both sexes rather than only one, and sex allocation theory predicts that the relative allocation toward the male and the female sex function depends on the individual's condition (often discussed in the context of size‐dependent sex allocation; Charnov 1982; Schärer 2009). This theory relies on the assumption that the male fitness gain curve (depicting the fitness return per investment) shows initially a steep increase, but then saturates (i.e., diminishing returns for any additional investment) as a consequence of an increased sperm competition between related sperm by the same donor (termed “local sperm competition”; Schärer 2009). By contrast, fitness through the female sex function is expected to increase linearly with increasing investment. Importantly, the male gain curve is expected to increase initially more steeply than the female one, leading to higher fitness returns for any initial investment in the male sex function. As a consequence, individuals in poor condition are predicted to invest relatively more resources in the male sex function compared with individuals in good condition (Schärer 2009). Such condition‐dependent sex allocation has been documented for the hermaphroditic flatworm *Macrostomum lignano* by showing that food‐restricted worms develop relatively smaller ovaries than well‐fed worms, suggesting a more male‐biased sex allocation of individuals in poor condition as expected by theory (Vizoso and Schärer 2007).

Condition‐dependent sex allocation will eventually affect condition dependence of male and female reproductive success. On the assumption of a more male‐biased sex allocation of individuals in poor condition, we predict that condition dependence of reproductive success is stronger for the female sex function even in the presence of intense male–male competition. We used a similar approach as taken by Zikovitz and Agrawal (2013) in order to explore sex‐specific condition dependence of reproductive success in the simultaneously hermaphroditic freshwater snail *Physa acuta*. Previous studies on *P. acuta* indicated that precopulatory sexual selection is typically stronger on the male sex function (Pélissié et al. 2012; but see Janicke et al. 2015a). Moreover, paternity analyses revealed intense sperm competition, suggesting that the male function is also subject to postcopulatory sexual selection in this species (Pélissié et al. 2014; Janicke et al. 2015a). In this study, we subjected focal snails to two feeding treatments (i.e., ad libitum food supply and short‐term starvation) and tested for condition dependence of male and female reproductive success under varying levels of male–male competition (i.e., monogamy and polygamy). Given the prediction derived from sex allocation theory, we expected that (1) selection on condition is generally stronger through the female compared with the male sex function, but that (2) this sex difference is weaker in the polygamy treatment compared with the monogamy treatment, as sexual selection promotes condition dependence of male reproductive success.

## Methods

### Study organism

We studied condition dependence of male and female fitness components in the simultaneously hermaphroditic freshwater snail *P. acuta* (Draparnaud 1805), which is a facultative outcrossing species. Individuals self‐fertilize their eggs only in the absence of mating partners (Jarne et al. 2000), as selfing is associated with substantial inbreeding depression in both sex functions (e.g., Janicke et al. 2013). Copulations are always unilateral (i.e., individuals adopt either the male or the female sex function) and are characterized by a stereotypic behavior by both partners. The male‐acting snail crawls on the shell of the partner trying to mount in a position that allows it to insert the phallus into the partner's gonopore (Wethington and Dillon 1996). By contrast, the female‐acting snail remains mainly passive, but can reject sperm donors by means of a so‐called “shell swinging” behavior (Wethington and Dillon 1996; Janicke et al. 2014). In the laboratory, snails are usually kept at 25°C in small plastic boxes (200 mL) and fed with boiled lettuce. Under these conditions, snails mature within 6–8 weeks and adults lay a gelatinous egg capsule containing several tens of eggs every 1–2 days.

### Experimental setup

#### Breeding of focal and albinotic individuals

Parents of wild‐type focal individuals were collected in April 2013 from a natural population near the spring of the river Lez (50°55.6′N, 11°34.6′E), located 15 km north of Montpellier, France. In total, we sampled 120 immature individuals in the field and kept them in isolation under laboratory conditions until maturity. On day one of the experiment, we pooled all grown adult individuals in one tank to let them copulate for 3 h. Afterward, we isolated all individuals to let them lay eggs for 3 days. This ensured that all resulting offspring were of similar age. On day 16, we pooled all offspring, sampled randomly 160 individuals (hereafter called “focals”), and raised them in isolation under ad libitum food conditions until maturity.

Albinotic snails served as potential mating partners and competitors of focal individuals. These snails are homozygous for a recessive, Mendelian segregating allele for albinism. Using albinotic snails as potential competitors enabled us to quantify male reproductive success of the wild‐type focal individual in a competitive context. The used albino culture line has been backcrossed three times into a genetically outbred population, so that all albinotic snails have an outbred genome except for the small region around the locus for albinism. Albinotic snails have been demonstrated not to differ from wild‐type individuals in terms of male and female mating behavior (Janicke et al. 2014) and reproductive success (Janicke et al. 2015a).

Albinotic individuals were bred in a similar way as focal individuals. Specifically, on day one, we pooled 120 adult albinotic individuals from the albino stock culture and let them copulate for 3 h. Next, we isolated all individuals for 4 days to lay eggs. On day 18, we pooled all resulting offspring and sampled randomly 400 juveniles (hereafter called “albinotic mates”). We kept all offspring in isolation, providing food ad libitum until we applied the experimental feeding and mating system treatments.

#### Experimental manipulation of food availability and mating system

We assessed male and female reproductive performance of focal snails under varying levels of food availability and sexual selection using a full‐factorial design. Specifically, we applied two feeding regimes that allowed us to test for condition dependence. In the High‐food treatment (hereafter “H‐treatment”), focals were provided food ad libitum throughout the experiment. In the Low‐food treatment (hereafter “L‐treatment”), the focal snail had no access to food for 4 days prior to the mating trials. Both well‐fed (H‐treatment) and poorly fed (L‐treatment) snails were subjected to two mating systems. In the enforced monogamy treatment (hereafter “M‐treatment”), focal individuals were paired with a single unmated albinotic snail, preventing pre‐ and postcopulatory sexual selection to operate. In the polygamy treatment (hereafter “P‐treatment”), focal individuals were grouped with four unmated albinotic individuals, imposing intense pre‐ and postcopulatory sexual selection (Pélissié et al. 2012, 2014; Janicke et al. 2015a). All albinotic mates were kept under ad libitum food conditions throughout the experiment.

The food manipulation and the mating trials were split over six consecutive days corresponding to six independent blocks. Every day from days 41 to 46, we transferred 16 focal snails, now mature in both sex functions, separately to boxes with fresh food (H‐treatment) and 16 focal snails to boxes without any food (L‐treatment). This feeding treatment was applied for a period of 4 days, during which all individuals remained virgin in both sex functions. One day before the mating trials, we measured body weight for all focal snails (measured to the nearest 0.1 mg using a Mettler PM100 balance; Mettler‐Toledo, Greifensee, Switzerland). On the same day, we also color‐marked all albinotic mating partners individually on their shell using Edding 751 gloss paint marker (Edding, Germany), which allowed us to identify all individuals during the mating trials. Previous studies indicated that color‐marked individuals do not differ from untreated individuals in terms of mating behavior and reproductive success (Henry and Jarne 2007; Janicke et al. 2014).

From days 45 to 50, we performed the mating trials, during which focal individuals were assigned to the mating system treatment. Specifically, randomly chosen focals of both feeding treatments were either grouped with one (M‐treatment) or four (P‐treatment) albinotic snails in glass beakers filled with 350 mL water for 3 h without providing food. During this period, we recorded the number of male and female copulations of all focal individuals. After the mating trials, all focal and albinotic individuals were isolated in boxes and fed ad libitum to lay eggs for 4 days.

In the above‐outlined experimental design, all individuals were initially unmated and grouped for a relatively short period of 3 h. This might have limited the effect of our food manipulation on reproductive performance, especially for the male sex function, as poorly fed, unmated sperm donors might have had accumulated sufficient autosperm in storage to perform equally well as well‐fed donors. In order to test whether our results are robust to an alternative setup in which all individuals are already mated and had to compete for a longer period, we repeated the food manipulation and the mating trials for each focal individual in the second experimental run. From days 49 to 54, we repeated the feeding treatment for 4 days as described for the first experimental run (see above) and measured the body weight of all focal snails 1 day before the mating trials. From days 53 to 58, all focals were grouped with their previous albinotic mates in plastic boxes (M‐treatment: 200‐mL boxes; P‐treatment or 400‐mL boxes) for 24 h, and provided food ad libitum. Afterward, all individuals were isolated in fresh boxes to lay eggs for 4 days. Therefore, the second experimental run differs from the first run in three aspects. First, all individuals were nonvirgins. Second, mating trials lasted for 24 h instead of only 3 h, imposing enduring pre‐ and postcopulatory male–male competition. Third, we did not record the mating behavior and only quantified body weight and reproductive success of both sex functions.

Focal and albinotic individuals were randomly assigned to all treatments, meaning that potential family effects due to our breeding protocol (see above) are balanced across all treatments and can therefore be neglected. Moreover, we balanced all treatments across the six blocks. Postexperimental tests showed that all measured traits were unaffected by block (all *P* > 0.05), so we did not include block effects in the statistical analysis.

Note that the experiment reported here was part of a bigger study comprising additional treatments. However, all reported data have not been published elsewhere, except the HP‐treatment, which was included in a test for environment‐dependent sexual selection published elsewhere (Janicke et al. 2015a).

#### Quantifying male and female reproductive performance

After 4 days of egg laying, we removed all focal and albinotic snails from the boxes and kept all egg capsules and the resulting offspring. On days 50–55 (second run: days 62–67), we counted all eggs and on days 56–61 (second run: days 68–73), we assessed the number of all hatched wild‐type and albinotic offspring produced by focals and albinotic mates. Male reproductive success was defined as the total number of wild‐type juveniles produced by all albinotic mates of the focal's social group. Female reproductive success was measured as the number of maternally produced (wild‐type) juveniles of the focal snail.

In order to obtain a more fine‐tuned understanding of condition dependence of male and female reproductive performance, we decomposed male and female reproductive success into main fitness components. Male reproductive success was decomposed into the number of copulations in the male role and the number of sired offspring per copulation, which provide estimates for pre‐ and postcopulatory reproductive performance, respectively. For this, we recorded the number of copulations in the male role for all focal snails. Mating effort in *P. acuta* is highly male biased, as the male‐acting individual spends energy for mate searching, shell mounting, and avoidance of rejection behavior (Jarne et al. 2010). Moreover, while mating, the male‐ but not the female‐acting snail is prevented from food searching and ingestion, which imposes an additional mating cost on the sperm donor. For these reasons, mating in the male role has been argued to serve as a good surrogate for male, but not female, reproductive investment (Auld et al. 2014; for a critical review of alternative proxies for male allocation in simultaneous hermaphrodites, see Schärer 2009). The number of offspring per copulation is expected to reflect the focal's sperm competitiveness, which is supposedly linked with investment in the production of sperm and/or seminal fluids.

Female reproductive success was partitioned into three fitness components, namely the number of copulations in the female function, the number of eggs produced, and hatching success (i.e., the number of hatched juveniles per egg). Notably the number of eggs is considered as a good proxy for female reproductive investment (Schärer 2009; Auld et al. 2014).

### Statistical analyses

Each treatment combination was initially replicated 40 times. However, two focals escaped from the boxes during growth (one individual of the HP‐treatment and one individual of the LP‐treatment), resulting in a total sample size of 158 focal individuals.

We fit generalized linear models (GLMs) to test for an effect of food availability and mating system on body weight and reproductive performance. Specifically, we modeled body weight and the number of sired offspring per copulation assuming a Gaussian error structure (identity link). Note that body weight was measured prior to the mating trials and was therefore not expected to differ between the mating systems. Nevertheless, we subjected body weight to a full model in order to verify that individuals were randomly assigned to both mating systems. The number of eggs, the number of copulations, reproductive success of both sex functions, and total reproductive success (i.e., sum of offspring produced through the male and female sex function) were modeled assuming a Poisson error structure (log‐link). Hatching success was modeled as a binomial variable (logit‐link).

We tested for sex‐specific condition dependence by modeling total reproductive success as a function of sex, food availability, and mating system using Linear Mixed‐Effects Models (LMMs) with focal individual defined as a random factor. Reproductive success was square root transformed to fulfill the assumption of normality. In these LMMs, the sex by food availability interaction indicates whether condition dependence of reproductive success is sex specific, whereas the three‐way interaction indicates whether sex‐specific condition dependence differs among the tested mating systems. For quantifying the strength of selection on condition, we computed the selection coefficient against poorly fed individuals following Zikovitz and Agrawal (2013). Specifically, we assessed the strength of selection on condition as *s *=* *1 – *W*_L_/*W*_H_, where *W*_L_ and *W*_H_ are the mean number of offspring produced by snails of the L‐ and H‐treatment, respectively. We computed *s* and its 95% confident limits separately for each sex function and mating system treatment using bootstrapping (10,000 bootstrap replicates). All statistical analyses were carried out in R version 3.2.0 (R Core Team 2015).

## Results

**Figure 1:**
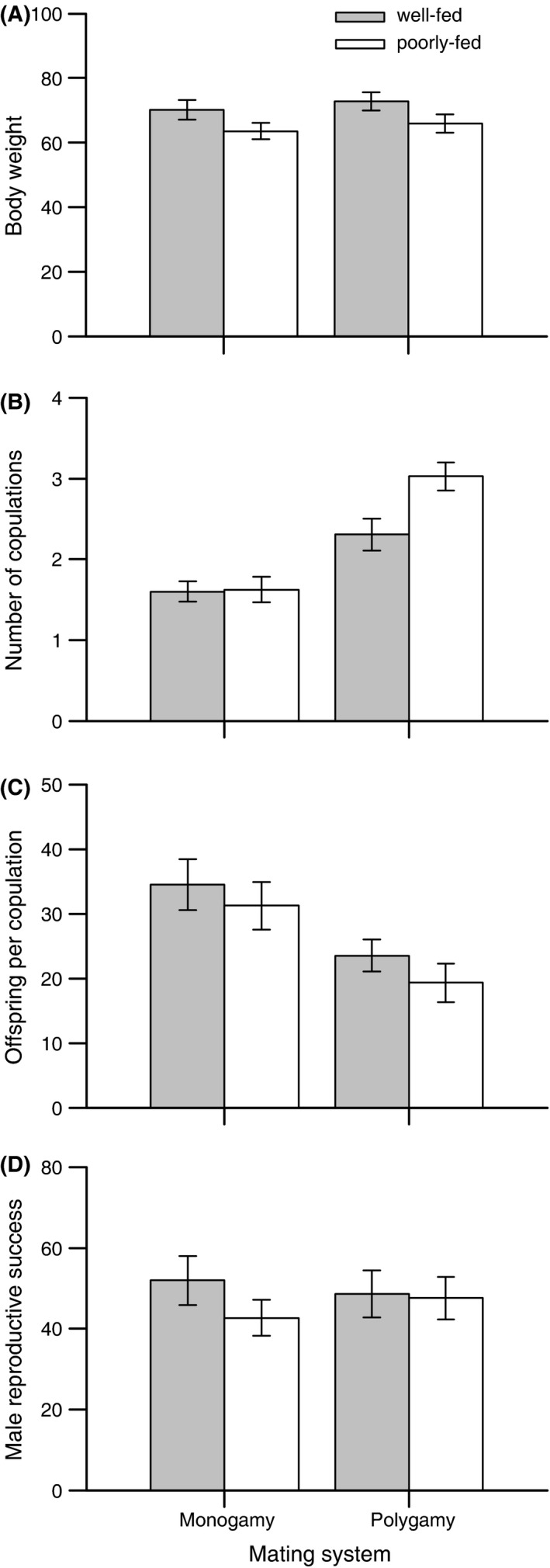
Effects of food availability and mating system on (A) body weight and male reproductive performance estimated as (B) the number of copulations in the male role, (C) the number of offspring per copulations, and (D) male reproductive success. Error bars show means ± 1 SE of well‐fed (filled bars) and poorly fed (open bars) snails.

**Figure 2:**
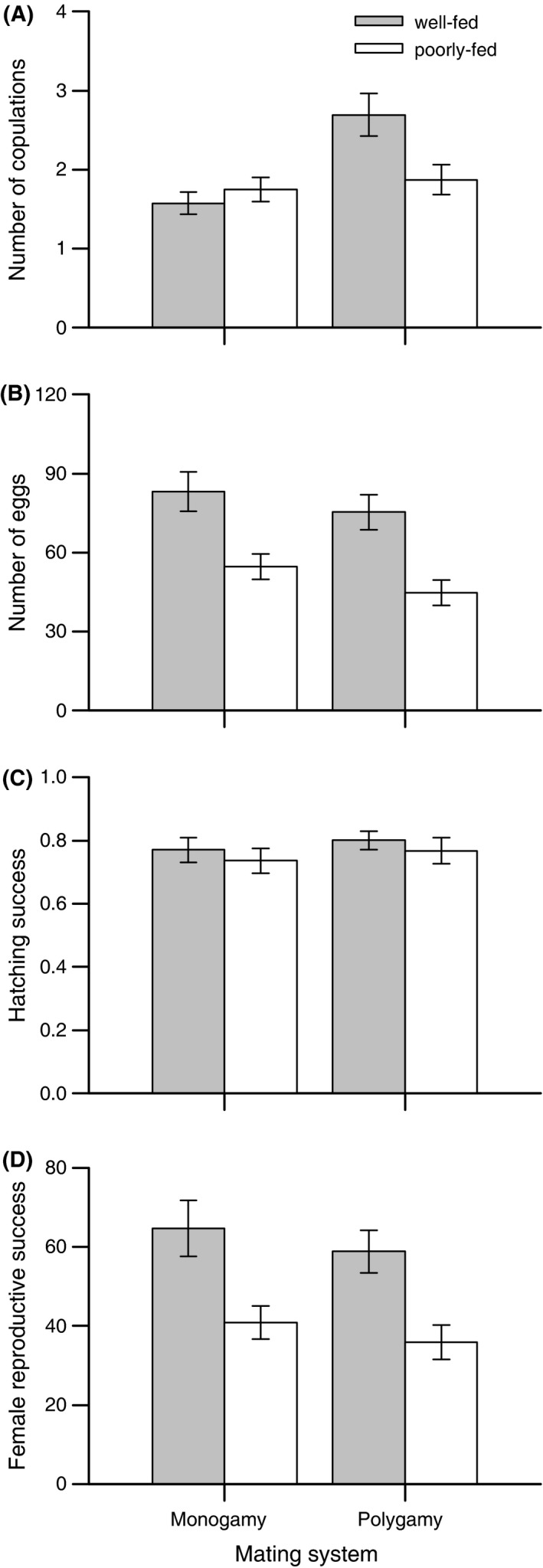
Effects of food availability and mating system on female reproductive performance estimated as (A) the number of copulations in the female role, (B) the number of eggs produced, (C) hatching success, and (D) female reproductive success. Error bars show means ± 1 SE of well‐fed (filled bars) and poorly fed (open bars) snails.

Food availability affected body weight such that poorly fed individuals were lighter than well‐fed individuals (Table 1; Fig. 1A). In the male role, poorly fed individuals copulated more often, but showed a tendency for obtaining less offspring per copulation compared with well‐fed individuals (Table 1; Fig. 1B and C). As a consequence, male reproductive success was not affected by the feeding treatment (Table 1; Fig. 1D). In the female role, poorly fed snails copulated less often in the polygamy, but not in the monogamy, treatment (Table 1; Fig. 2A; Tukey post‐hoc tests, P‐treatment: *z* = 2.825, *P *=* *0.024, M‐treatment: *z *=* *−0.718, *P* = 0.889). Moreover, poorly fed snails laid fewer eggs, but did not show a reduced hatching success (Table 1; Fig. 2B and C), leading to a reduced female reproductive success compared with well‐fed snails (Table  1; Fig. 2D). These effects on the female sex function resulted in a lower total reproductive success of poorly fed individuals (Table 1; pooled means ± SE, well‐fed: 112.14 ± 6.42, poorly fed: 83.48 ± 4.83). Effects of food availability on female and total reproductive success were not just a consequence of a lower body weight of poorly fed individuals, but remained significant after controlling statistically for differences in body weight (GLMs with body weight included as a covariate; female reproductive success, food availability: *F*_1,153_ **=* *6.627, *P *=* *0.011, mating system: *F*_1,153_ **=* *1.437, *P *=* *0.232, food availability × mating system: *F*_1,153_ **=* *0.058, *P *=* *0.811, body weight: *F*_1,153_ **=* *45.141, *P *<* *0.001; total reproductive success, food availability: *F*_1,153_ **=* *5.642, *P *=* *0.019, mating system: *F*_1,153_ **=* *0.062, *P *=* *0.804, food availability × mating system: *F*_1,153_ **=* *0.257, *P *=* *0.613, body weight: *F*_1,153_ **=* *24.710, *P *<* *0.001).

**Table 1:** Effect of food availability and mating system on body weight and reproductive performance in *P. acuta*. Generalized linear models are shown separately for shared fitness components, male reproductive performance, and female fitness components measured in the first experimental run. Significant effects (*P *<* *0.05) are highlighted in boldface

Response	Food availability treatment			Mating system treatment			Food availability × Mating system		
	df	*F*	*P*	df	*F*	*P*	df	*F*	*P*
(a) Shared fitness component									
Body weight	1,156	5.930	**0.016**	1,155	0.789	0.376	1,154	0.005	0.947
Total reproductive success	1,156	12.932	**<0.001**	1,155	0.341	0.560	1,154	0.237	0.627
(b) Male fitness components									
Number of copulations	1,156	4.864	**0.029**	1,155	40.386	**<0.001**	1,154	2.511	0.115
Number of offspring per copulation	1,156	1.464	0.228	1,155	11.993	**0.001**	1,154	0.022	0.884
Male reproductive success	1,156	0.947	0.332	1,155	0.020	0.889	1,154	0.602	0.439
(c) Female fitness components									
Number of copulations	1,156	2.822	0.095	1,155	10.832	**0.001**	1,154	5.808	**0.017**
Number of eggs	1,156	24.943	**<0.001**	1,155	2.225	0.138	1,154	0.285	0.595
Hatching success	1,141	0.051	0.821	1,140	0.436	0.510	1,139	0.499	0.481
Female reproductive success	1,156	19.937	**<0.001**	1,155	1.055	0.306	1,154	0.028	0.868

**Figure 3:**
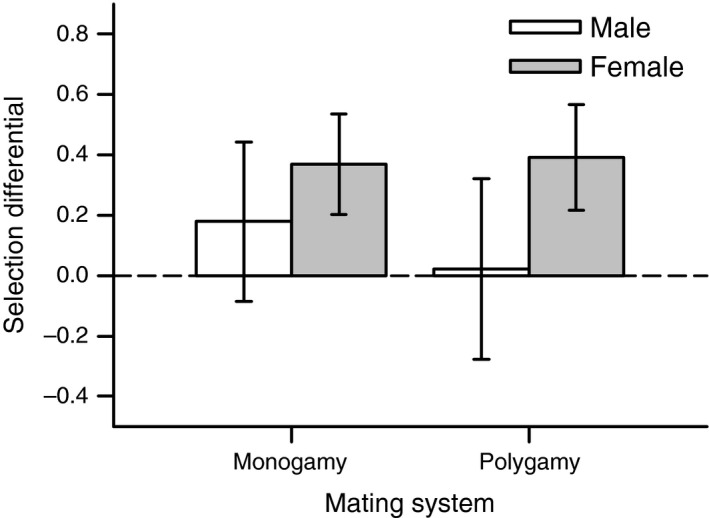
Selection on condition shown for the male (open bars) and female (filled bars) sex function subjected to two mating systems. Selection coefficients quantifies the strength of selection against poorly fed individuals (for details see ‘Methods’ section). Error bars show means ± 95% CI.

Condition dependence of reproductive success differed between both sex functions, with stronger selection against poorly fed individuals in the female sex function (Table 2; Fig. 3). This sex‐specific condition dependence was not affected by the mating system (Table 2). All results on condition dependence of body weight and reproductive performances could be confirmed by the second experimental run (Tables S1 and S2; Figs S1–S3).

**Table 2:** Effects of food availability, mating system, and sex on the total number of offspring produced (i.e., number of paternally plus maternally produced offspring) measured in the first experimental run. Summary statistics of a Linear Mixed‐Effects Model with focal individual defined as a random factor are shown. Significant effects (*P *<* *0.05) are highlighted in boldface

Predictor	Analysis of variance		
	df	*F*	*P*
Food availability	154	7.771	**0.006**
Mating system	154	0.071	0.791
Sex	154	0.194	0.661
Food availability × Mating system	154	0.038	0.846
Food availability × Sex	154	6.067	**0.015**
Mating system × Sex	154	2.052	0.154
Food availability × Mating system × Sex	154	0.805	0.371

## Discussion

This study provides the first experimental test for sex‐specific condition dependence of reproductive success in a simultaneous hermaphrodite. We found that food limitation impaired female, but not male, reproductive success, suggesting that selection on condition is stronger through the female sex function even under conditions imposing pre‐ and postcopulatory sexual selection. Interestingly, poorly fed snails showed an increased male copulatory activity, suggesting that food availability affected sex allocation of focal individuals. In the following, we discuss these main findings in the context of condition‐dependent sex allocation and allude to its implications for the evolution of sexually selected traits in simultaneous hermaphrodites.

### Condition dependence of male and female fitness components

Our experimental manipulation of food availability successfully induced variation in condition of focal individuals, as indicated by a reduced weight and a lowered total reproductive success of poorly fed individuals. Food restriction diminished total reproductive success solely as a consequence of a lowered number of offspring produced by the female sex function, which itself was primarily driven by condition‐dependent egg production. By contrast, male reproductive success remained unaffected by our feeding treatment in both mating system treatments, suggesting that condition does not affect male reproductive output irrespective of the intensity of male–male competition.

Condition dependence of body weight and female fecundity is evident in separate‐sexed organisms (e.g., Selman and Houston 1996; Warner et al. 2007) and has also been demonstrated for several simultaneous hermaphrodites including *P. acuta* (e.g., Janicke et al. 2010; Graham et al. 2015). The more striking finding of our study is the observation that condition does not affect the number of offspring produced through the male sex function. This stands in stark contrast to studies in separate‐sexed organisms that found evidence for condition dependence of male reproductive success (e.g., Fricke et al. 2008; Zikovitz and Agrawal 2013). We suspect that the observed lack of condition dependence of male reproductive success in *P. acuta* is caused by an increased male allocation in poorly fed individuals, as predicted by sex allocation theory for simultaneous hermaphrodites (reviewed in Charnov 1982; Schärer 2009). Poorly fed individuals clearly suffered from an overall smaller pool of resources, but copulated more often in the male role, suggesting that they allocated relatively (but not absolutely) more resources to the male sex function. Thereby, poorly fed individuals might have compensated an adverse effect of food restriction on other male fitness components. Assuming a fixed amount of available resources, this increased male investment in mate acquisition must have been traded off with investment in other fitness components such as other male traits (e.g., production of sperm and/or seminal fluid), somatic growth or maintenance, and the allocation toward the female sex function. Hence, at least part of the observed reduction in body weight and/or egg production of poorly fed individuals can be attributed to a higher investment in male mate acquisition.

Quantifying sex allocation in simultaneous hermaphrodites is a challenging task (reviewed in Schärer 2009), and for *P. acuta* it remains questionable whether investment in male mating frequency serves as a good proxy for total male reproductive investment or whether it trades off with other male fitness components. However, the number of male copulations can definitely be considered as an investment in the male sex function, as it is always the sperm donor who initiates the copulation, which is likely to be costly in terms of energy and time (Auld et al. 2014). We also observed a reduced female copulation frequency in poorly fed individuals. However, we believe that this does not necessarily indicate a reduced allocation toward the female function, because female‐acting snails do not actively search for partners and do not initiate a copulation. Instead, we speculate that the reduced female mating success of poorly fed focals reflects mate choice against poorly fed (and thus less fecund) female mating partners, as already documented for *P. acuta* (Ohbayashi‐Hodoki et al. 2004) and other simultaneous hermaphrodites (e.g., Janicke et al. 2012). This is supported by the observation that the effect of food availability on copulation frequency was not observed in pairs in which there was no scope for mate choice.

Our finding of a female‐biased condition dependence of reproductive success is consistent with sex allocation theory predicting a more male‐biased sex allocation of individuals in poor condition. Nevertheless, there are at least three alternative explanations for the observed lack of male condition dependence. First, our experimental conditions might have failed to impose male–male competition, allowing poorly fed sperm donors to perform equally well as well‐fed donors. This hypothesis seems unlikely, as previous studies on *P. acuta* using exactly the same experimental setup as our polygamy treatment (i.e., focal individual grouped together with four well‐fed snails) revealed substantial pre‐ and postcopulatory sexual selection in terms of larger variances in mating success and steeper Bateman gradients in the male compared with the female sex function (Pélissié et al. 2012, 2014; Janicke et al. 2015a). Second, our experimental conditions might have failed to constrain male reproductive success because mating trials of 3 h might have been too short to deplete autosperm reserves, so that poorly fed snails performed equally well in their male role as well‐fed snails. However, we obtained qualitatively the same results from a second experimental run in which all focal individuals were subjected to mating trials that lasted 24 h. This suggests that our results are not sensitive to the time period that focal individuals competed for the eggs of their group members. Finally, our food manipulation of 4 days might have been too short to induce a significant effect on male reproductive success, especially because similar studies on separate‐sexed organisms manipulated food availability for longer periods (e.g., Fricke et al. 2008; Zikovitz and Agrawal 2013). However, our food manipulation was definitely effective in inducing variation in condition of focal individuals (see above). This was probably due to the fact that we provided no food in the Low‐food treatment rather than less food or food of a lower nutritional value, as has been carried out in the other studies. Importantly, the aim of our feeding treatment was to manipulate the condition of focal individuals at a moderate level to an extent that does not provoke a complete reproductive failure, allowing us to test for sex‐specific condition dependence. We presume that a more stringent food manipulation will at some point also affect male reproductive success and further studies should quantify male and female reproductive performance under more variable feeding regimes in order to obtain a more nuanced understanding of sex‐specific condition dependence of reproductive success in *P. acuta*.

### Implications for sexual selection in simultaneous hermaphrodites

Sexual selection has repeatedly been argued to be less intense in simultaneous hermaphrodites as exaggerated secondary sexually selected traits are virtually lacking in this group of organisms (reviewed in Anthes 2010; Schärer et al. 2015). Darwin already claimed that hermaphrodites “have too imperfect senses and much too low mental powers to appreciate each other's beauty” (Darwin 1871) and also more recent theoretical work questioned the role of precopulatory sexual selection for the evolution of reproductive characters. For instance, the expression of costly male‐beneficial traits is likely to entail costs for the female sex function without conferring any benefit, which has been argued to impede the evolution of male ornaments or armaments in hermaphrodites (Morgan 1994). Moreover, having both sexes united in the same individual means that all individuals in the population search for a mating partner, whereas in separate‐sexed organisms, only individuals of the sex that benefit from mating (typically males; Janicke et al. 2015b) are expected to invest in mate acquisition. This implies that hermaphroditic individuals are more likely to be searched out by their partners and are generally more willing to mate if copulations involve reciprocal sperm transfer, which both of which are predicted to reduce selection for secondary sexually selected traits (Greeff and Michiels 1999).

Here, we hypothesize that condition‐dependent sex allocation provides an additional mechanism that hampers the evolution of sexually selected traits in simultaneous hermaphrodites. This is because condition‐dependent sex allocation may decouple the link between the expression of a costly male‐beneficial ornament or armament and the condition of its bearer. Specifically, hermaphroditic individuals in poor condition (i.e., those with an inferior resource acquisition) may express an ornament or armament of a similar size as individuals in a good condition just because the former are likely to channel relatively more resources toward the male function than the latter, so that the absolute investment in the male function can be equal for both. This relaxes the core assumption of the genic capture hypothesis, which is that sexually selected traits capture the overall genetic quality and thus the condition of an individual (Rowe and Houle 1996). Moreover, condition‐dependent sex allocation implies that a costly male‐beneficial trait may constitute an unreliable indicator for the signaler's genetic quality, impeding the evolution of handicap signals by mate choice (Zahavi 1975; Maynard Smith and Harper 1995). This scenario holds as long as individuals in poor condition still have enough resources to reallocate to the male function, so that their absolute male allocation is similar compared with individuals with a better condition.

Explicit theoretical work is certainly needed to really understand how condition‐dependent sex allocation can affect the evolution of sexually selected traits in hermaphrodites. This should include models that explore the evolution of costly male‐beneficial traits under varying sets of male and female fitness gain curves. Furthermore, we still know very little about condition dependence of sexually selected traits in simultaneous hermaphrodites. There are a few male traits that have been demonstrated to be under sexual selection in hermaphrodites such as the male copulatory organ (Janicke and Schärer 2009; Garefalaki et al. 2010) and the so‐called love darts (e.g., Chase and Blanchard 2006). However, we are not aware of any experimental study testing for condition dependence of these traits. Our study revealed a higher male mating rate of individuals in poor condition, but the underlying traits determining male mating success still need to be identified in order to test for condition dependence of sexually selected traits in *P. acuta*.

To close, we note that condition‐dependent allocation toward sexually selected traits has also been studied in separate‐sexed organisms (Houslay and Bussiere 2012; Morehouse 2014). There are a few examples indicating that males in poor condition increase their investment in sexually selected traits at the expense of longevity. For instance, food‐restricted males of the cricket *Gryllus assimilis* exhibit higher investment in female attraction by increasing their calling effort (Whattam and Bertram 2011). The authors argue that males in poor condition are facing a lower future reproductive potential compared with males in good condition and therefore increase their investment in current reproduction to maximize fitness. Similar results have been documented for other cricket species (Hunt et al. 2004; Houslay et al. 2015) and fish (Candolin 1999). However, as true for simultaneous hermaphrodites, the implications of such condition‐dependent life‐history allocation for the evolution of sexually selected traits have rarely been explored in separate‐sexed organisms (but see Kokko 1997), highlighting the need for more theoretical work on this topic.

## Conclusions

This study provides the first experimental test of sex‐specific condition dependence of reproductive success in a simultaneous hermaphrodite. We found that female, but not male, reproductive success was affected by the applied feeding treatment, suggesting that selection on condition is stronger through female sex function. We suspect that the observed effects are driven by condition‐dependent allocation of resources toward the male and female sex function, as pre dicted by sex allocation theory. Our findings have important implications for the evolution of costly sexually selected traits and we stress the need for more theoretical and empirical work on condition dependence of male fitness components in simultaneous hermaphrodites.

## Conflict of Interest

None declared.

## Supporting information


**Table S1**. Effect of food‐availability and mating system on body weight and reproductive performance in *P. acuta*.
**Table S2**. Effects of food availability, mating system and sex on the total number of offspring produced (i.e., number of paternally plus maternally produced offspring) measured in the second experimental run.
**Figure S1**. Schematic illustration of the experimental setup.
**Figure S2**. Effects of food availability and mating system on (A) body size, (B) male and (C) female reproductive success.
**Figure S3**. Selection on condition of the male (open bars) and female (filled bars) sex function subjected to two mating systems.
