# Supplementary material for: Condition dependence of male and female reproductive success: insights from a simultaneous hermaphrodite
Source: Ecol Evol. 2016 Jan 18;6(3):830–41. doi: 10.1002/ece3.1916 (PMC4739575; doi:10.1002/ece3.1916)
Supplement: Supplementary file 1 — Table S1. Effect of food‐availability and mating system on body weight and reproductive performance in P. acuta. Table S2. Effects of food availability, mating system and sex on the total number of offspring produced (i.e., number of paternally plus maternally produced offspring) measured in the second experimental run. Figure S1. Schematic illustration of the experimental setup. Figure S2. Effects of food availability and mating system on (A) body size, (B) male and (C) female reproductive success. Figure S3. Selection on condition of the male (open bars) and female (filled bars) sex function subjected to two mating systems. [file ECE3-6-830-s001.docx]

# Supplementary Tables

Table S1. Effect of food-availability and mating system on body weight and reproductive performance in *P. acuta*. Generalized linear models are shown separately for shared fitness components, male reproductive performance and female fitness components measured in the second experimental run. Significant effects (*P* < 0.05) are highlighted in boldface.

|  | Food availability treatment | | | Mating system treatment | | | Food availability × Mating system | | |
| --- | --- | --- | --- | --- | --- | --- | --- | --- | --- |
|  | *dfs* | *F* | *P* | *dfs* | *F* | *P* | *dfs* | *F* | *P* |
| Body weight | 156 | 24.252 | **< 0.001** | 155 | 1.812 | 0.180 | 154 | 0.004 | 0.949 |
| Total reproductive success | 156 | 12.976 | **< 0.001** | 155 | 0.187 | 0.666 | 154 | 0.003 | 0.955 |
| Male reproductive success | 156 | 2.219 | 0.138 | 155 | 0.568 | 0.452 | 154 | 0.001 | 0.979 |
| Female reproductive success | 156 | 23.075 | **< 0.001** | 155 | 0.088 | 0.768 | 154 | 0.000 | 0.986 |

Table S2. Effects of food availability, mating system and sex on the total number of offspring produced (i.e., number of paternally plus maternally produced offspring) measured in the second experimental run. Summary statistics of a Linear Mixed-Effects Model with focal individual defined as a random factor are shown. Significant effects (*P* < 0.05) are highlighted in boldface.

|  | Analysis of variance | | |
| --- | --- | --- | --- |
| Predictor | *dfs* | *F* | *P* |
| Food availability | 154 | 15.336 | **< 0.001** |
| Mating system | 154 | 0.002 | 0.964 |
| Sex | 154 | 76.915 | **< 0.001** |
| Food availability x Mating system | 154 | 0.003 | 0.957 |
| Food availability x Sex | 154 | 10.117 | **0.002** |
| Mating system x Sex | 154 | 0.002 | 0.964 |
| Food availability x Mating system x Sex | 154 | 0.207 | 0.650 |

# Supplementary Figure Legends

Figure S1. Schematic illustration of the experimental setup. Note that after the first experimental run we subjected all individuals (with most of them already mated in both sex functions) to experimental steps (3) to (5) in a second experimental run.

Figure S2. Effects of food availability and mating system on (A) body size, (B) male and (C) female reproductive success. Results of the second experimental run are shown. Error bars show means ± 1 SE of well-fed (filled bars) and poorly-fed (open bars) snails.

Figure S3. Selection on condition of the male (open bars) and female (filled bars) sex function subjected to two mating systems. Results of the second experimental run are shown. Selection coefficient *s* quantifies the strength of selection against poorly-fed individuals (for details see Method section). Error bars show means ± 95 % CI.

# Supplementary Figures

Figure S1.

Figure S2.

Figure S3.
